# Extracellular Vesicles Do Not Mediate the Anti-Inflammatory Actions of Mouse-Derived Adipose Tissue Mesenchymal Stem Cells Secretome

**DOI:** 10.3390/ijms22031375

**Published:** 2021-01-29

**Authors:** María Carmen Carceller, María Isabel Guillén, María Luisa Gil, María José Alcaraz

**Affiliations:** 1 Interuniversity Research Institute for Molecular Recognition and Technological Development (IDM), Polytechnic University of Valencia, Av. Vicent A. Estellés s/n, Burjasot, 46100 Valencia, Spain; carmen.carceller@gmail.com (M.C.C.); isabel.guillen@uv.es (M.I.G.); 2 Department of Pharmacy, Faculty of Health Sciences, Cardenal Herrera-CEU University, Alfara del Patriarca, 46115 Valencia, Spain; 3 Institute of Biotechnology and Biomedicine, Department of Microbiology and Ecology, University of Valencia, Dr. Moliner 50, Burjasot, 46100 Valencia, Spain; M.Luisa.Gil@uv.es

**Keywords:** mouse-derived adipose tissue, mesenchymal stem cells secretome, extracellular vesicles, inflammation, macrophage

## Abstract

Adipose tissue represents an abundant source of mesenchymal stem cells (MSC) for therapeutic purposes. Previous studies have demonstrated the anti-inflammatory potential of adipose tissue-derived MSC (ASC). Extracellular vesicles (EV) present in the conditioned medium (CM) have been shown to mediate the cytoprotective effects of human ASC secretome. Nevertheless, the role of EV in the anti-inflammatory effects of mouse-derived ASC is not known. The current study has investigated the influence of mouse-derived ASC CM and its fractions on the response of mouse-derived peritoneal macrophages against lipopolysaccharide (LPS). CM and its soluble fraction reduced the release of pro-inflammatory cytokines, adenosine triphosphate and nitric oxide in stimulated cells. They also enhanced the migration of neutrophils or monocytes, in the absence or presence of LPS, respectively, which is likely related to the presence of chemokines, and reduced the phagocytic response. The anti-inflammatory effect of CM may be dependent on the regulation of toll-like receptor 4 expression and nuclear factor-κB activation. Our results demonstrate the anti-inflammatory effects of mouse-derived ASC secretome in mouse-derived peritoneal macrophages stimulated with LPS and show that they are not mediated by EV.

## 1. Introduction

Mesenchymal stem cells (MSC) are multipotent cells actively participating in repair processes. They migrate to the site of injury and cooperate with different types of stromal and inflammatory cells to repair tissues [1,2]. Therefore, MSC studies have revealed promising applications in tissue regeneration. They can be isolated from multiple tissue sources including bone marrow, adipose tissue, dental pulp, liver, muscle, umbilical cord blood, etc., [3,4]. Besides their differentiation and homing abilities, these cells modulate the local environment and activate endogenous progenitor cells [5]. MSC possess a variety of immunomodulatory properties which can be critical for their efficiency in regenerative medicine applications, and may also lead to the development of treatments for autoimmune disorders [3,6]. Therefore, the actions of MSC on immune responses and the possible mechanisms involved are under intense investigation [7,8,9,10]. The interaction of MSC with different immune cells may be dependent on cell contact and the release of different factors. A wide range of evidence indicates that MSC release bioactive molecules to induce autocrine or paracrine effects [4]. Recent investigations suggest that the anti-inflammatory and immunoregulatory effects of MSC may be mediated by their secretome, including soluble factors and extracellular vesicles (EV) [11,12,13]. Exosomes (EX) or small EV are a sub-type of EV with a size from 30 to 150 nm which arise from the endosomal cell compartment [14], whereas microvesicles (MV) or large EV have a diameter of 100–1000 nm and are released by ectocytosis of the plasma membrane [15]. Some studies suggest that EV released from MSC may have equal therapeutic effects that parent cells, and thus EV represent a novel therapeutic approach in regenerative medicine as an alternative to stem cell-based therapy [16].

Adipose tissue represents an abundant source of MSC for therapeutic applications [17]. Although MSCs may differ in their functional potential according to their tissue of origin [18], adipose tissue-derived MSC (ASC) share regenerative and immunomodulatory properties with other MSC [19]. We have previously reported that EV mediate the cytoprotective effects of conditioned medium (CM) from human ASC in different cell types, such as osteoblasts and chondrocytes from osteoarthritic patients [20,21]. We also found that ASC from mouse perigonadal fat downregulated early events of the zymosan-injected mouse air pouch model and these effects may be mediated by ASC secretome [22]. Nevertheless, the contribution of EV and soluble fractions to the anti-inflammatory effects of these cells is not known. The current study was aimed at improving our knowledge of the anti-inflammatory properties of mouse-derived ASC secretome. Therefore, we have investigated the influence of ASC CM and its fractions, MV, EX, and EV-free CM (CM-EV) on the response of mouse-derived peritoneal macrophages against lipopolysaccharide (LPS) in the early stages of the immune reaction.

## 2. Results

### 2.1. Characterization of EV

**Figure 1:**
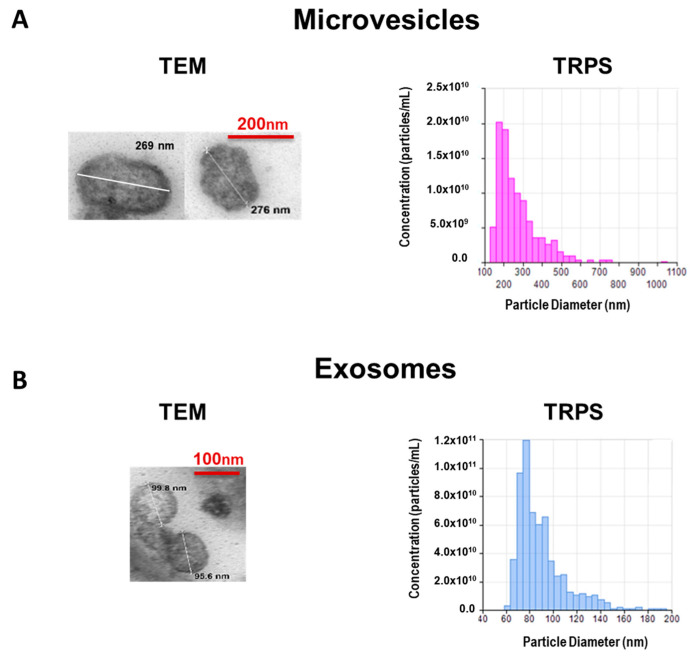
Characterization of microvesicles (**A**) and exosomes (**B**) isolated from CM of AMSC. Representative transmission electron microscopy (TEM) images with estimated size. Microparticle size distribution and concentration were determined by tunable resistive pulse sensing (TRPS).

CM was obtained from mouse-derived ASC as indicated in Materials and methods. EV were isolated from CM using differential centrifugation combined with size filtration. To study the morphology of EV we used transmission electron microscopy (TEM) as shown in Figure 1.

The concentration and size distribution were determined by the tunable resistive pulse sensing (TRPS) technology with the qNano instrument (IZON Science Ltd., Oxford, UK). MV showed an average size of 271 nm and a concentration of 1 × 10^10^ particles/mL (Figure 1A) while EX had an average size of 90 nm and a concentration of 6 × 10^11^ particles/mL (Figure 1B).

### 2.2. Release of Pro-Inflammatory Mediators

**Figure 2:**
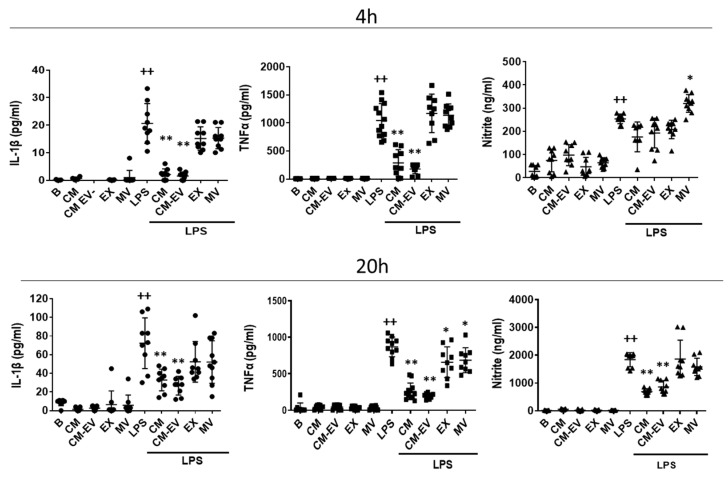
Release of pro-inflammatory mediators by mouse-derived peritoneal macrophages. Cytokines were measured by ELISA and nitrite was determined by fluorometry in cell supernatants at 4 h and 20 h of incubation with CM, CM-EV, EX, or MV in the presence or absence of LPS. B: cells incubated with control medium and not stimulated with LPS. Data are presented as mean ± SD (*n* = 9) from 3 independent experiments. One-way analysis of variance followed by Tukey’s post hoc test; ++ *p* < 0.001 versus B; * *p* < 0.05, ** *p* < 0.01 versus LPS control.

Mouse-derived peritoneal macrophages were stimulated with LPS for either 4 h or 20 h to study the effects of CM and its fractions on the release of pro-inflammatory mediators. In preliminary experiments, we carried out dose-response assays to select the EV concentrations showing the highest effect on the release of pro-inflammatory mediators without affecting cell viability assessed by the 3-(4,5- dimethylthiazol-2-yl)-2,5-diphenyl tetrazolium bromide (MTT) assay (data not shown). These concentrations (9 × 10^4^ particles/mL for MV and 2 × 10^7^ particles/mL for EX) were used in all experiments. Interleukin-1β (IL-1β) and tumor necrosis factor-α (TNFα) were measured in cell supernatants by ELISA. LPS stimulation enhanced the production of IL-1β and TNFα which was strongly reduced by CM and CM-EV at both incubation times. In comparison with these effects, MV or EX only exerted a weak reduction in TNFα secretion at 20 h (Figure 2). The content of nitrite in supernatants of macrophages stimulated with LPS was low at 4 h and these levels were not modified by the treatments except for a small increase induced by MV. In contrast, the strong production of nitrite induced by LPS at 20 h was significantly reduced by CM and CM-EV whereas EX and MV did not exert any effect.

### 2.3. Cell Migration

**Figure 3:**
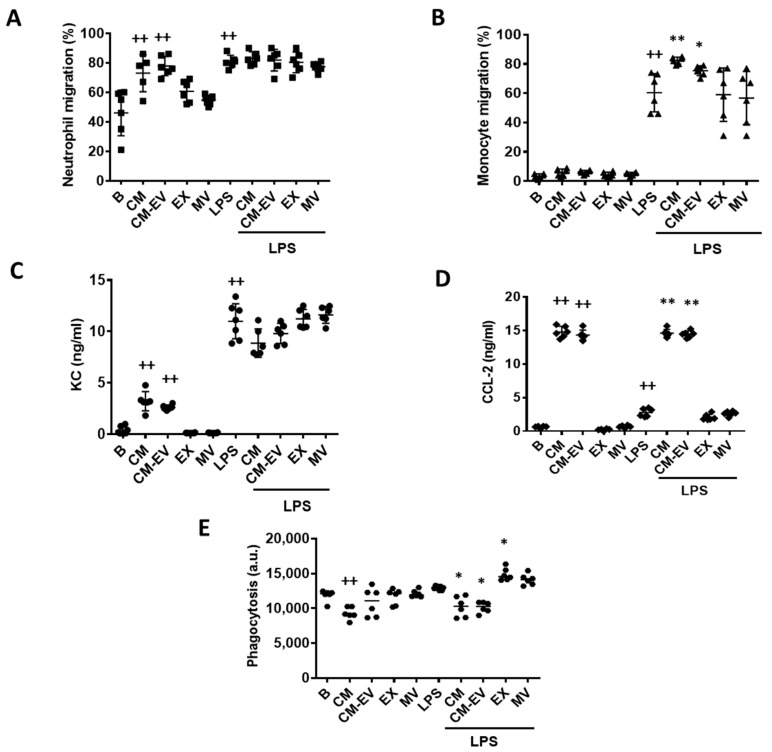
Macrophage functions. Neutrophil migration (**A**), monocyte migration (**B**), CXCL-1 levels (**C**), CCL-2 levels (**D**) and phagocytosis (**E**). For migration experiments, macrophages were incubated with CM, CM-EV, EX, or MV in the presence or absence of LPS for 20 h. Then, neutrophils or monocytes were added to the upper compartment of the transwells and after 4 h, the migrated cells were quantified by flow cytometry. CXCL-1 and CCL-2 were measured by ELISA in the supernatants of neutrophil or monocyte migration assays, respectively. Macrophage phagocytosis of fluorescent polystyrene beads was determined by flow cytometry. B: cells incubated with control medium and not stimulated with LPS. Data are presented as mean ± SD (*n* = 6) from 3 independent experiments. One-way analysis of variance followed by Tukey’s post hoc test; ++ *p* < 0.001 versus B; * *p* < 0.05, ** *p* < 0.01 versus LPS control.

The functional consequences of treating macrophages with CM or its fractions were next assessed. Therefore, we examined the effects of CM, CM-EV, EX, or MV treatment of macrophages on neutrophil and monocyte migration using transwell migration assays. In the first set of experiments, macrophages were incubated with CM, CM-EV, or EV in the presence or absence of LPS for 20 h. Then, neutrophils isolated from bone marrow were added into the inserts and after 4 h, the migrated neutrophils were determined by flow cytometry. As shown in Figure 3A, CM and CM-EV significantly enhanced neutrophil migration compared to the control group in the absence of LPS stimulation of macrophages whereas EX or MV did not modify the number of migrated cells. In contrast, neutrophil migration stimulated by LPS was not significantly modified by any of the treatments.

In the second set of experiments, we examined the migration of monocytes. Macrophages were treated as above, and monocytes isolated from bone marrow were then added into the inserts. After 4 h, the migrated monocytes were determined by flow cytometry. Figure 3B shows that none of the treatments affected monocyte migration in the absence of LPS. This stimulus significantly enhanced monocyte migration and treatment with either CM or CM-EV further increased it compared to the LPS control group.

It is known that MSC secrete chemokines such as (C-X-C motif) ligand 1 (CXCL-1) and C-C motif chemokine ligand (CCL)-2 [23]. CM or CM-EV provides high levels of these chemokines as measured in the supernatant of migration experiments (Figure 3C,D). Besides, LPS stimulation significantly enhanced the release of CXCL-1 and CCL-2 compared to unstimulated control cells whereas EX or MV treatment did not modify chemokine levels either in the presence or absence of LPS.

### 2.4. Phagocytic Activity

One of the main functions of macrophages in the innate inflammatory response is phagocytosis to remove pathogens, cell debris, and foreign particles [24]. The phagocytic activity of macrophages was assessed by using fluorescent beads. For this purpose, macrophages were treated with CM, CM-EV, EX, or MV in the presence or absence of LPS for 20 h. Fluorescent beads were added into the wells for 4 h, and the phagocytic activity was measured by flow cytometry. As shown in Figure 3E, CM treatment significantly reduced the phagocytosis of fluorescent beads by macrophages either in unstimulated cells or in the presence of LPS. CM-EV also decreased phagocytosis in LPS-stimulated macrophages while MV and EX were ineffective in unstimulated cells and increased the phagocytic activity of LPS-stimulated macrophages.

### 2.5. Expression of Cluster of Differentiation 14 (CD14) and Toll-Like Receptor 4 (TLR4)

**Figure 4:**
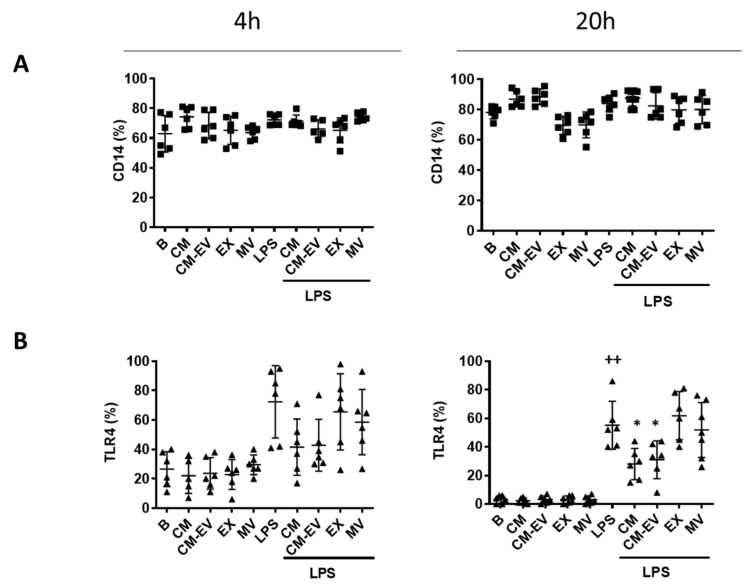
Expression of CD14 (**A**) and TLR4 (**B**). Macrophages were incubated with CM, CM-EV, EX, or MV in the presence or absence of LPS for 4 h or 20 h. Protein expression of CD14 and TLR4 was determined by flow cytometry. B: cells incubated with control medium and not stimulated with LPS. Data are presented as mean ± SD (*n* = 6) from 3 independent experiments. One-way analysis of variance followed by Tukey’s post hoc test; ++ *p* < 0.001 versus B; * *p* < 0.05 versus LPS control.

The stimulation of TLR4 by LPS induces the internalization of the receptor followed by the release of critical pro-inflammatory cytokines that are necessary to activate potent immune responses [25]. CD14 transfers LPS to TLR4, acting as an accessory protein [26]. We investigated if CD14 expression on macrophages may be affected by CM or its fractions in the presence or absence of LPS at two different time points (4 h and 20 h). As shown in Figure 4A, there were no significant changes in CD14 expression on the surface of macrophages. We wondered whether the anti-inflammatory effects of CM may be dependent on the regulation of TLR4 expression. TLR4 protein expression on macrophages was determined by flow cytometry at 4 h and 20 h after LPS stimulation. Figure 4B shows that LPS enhanced the expression of TLR4 at both time points compared to unstimulated control cells although the results were significant at 20 h only. We observed that none of the treatments modified the expression of TLR4 on the surface of macrophages at 4 h. In contrast, TLR4 was significantly downregulated by CM and CM-EV in cells stimulated with LPS for 20 h whereas EX and MV were ineffective.

### 2.6. Nuclear Factor κB (NF-κB) Translocation

**Figure 5:**
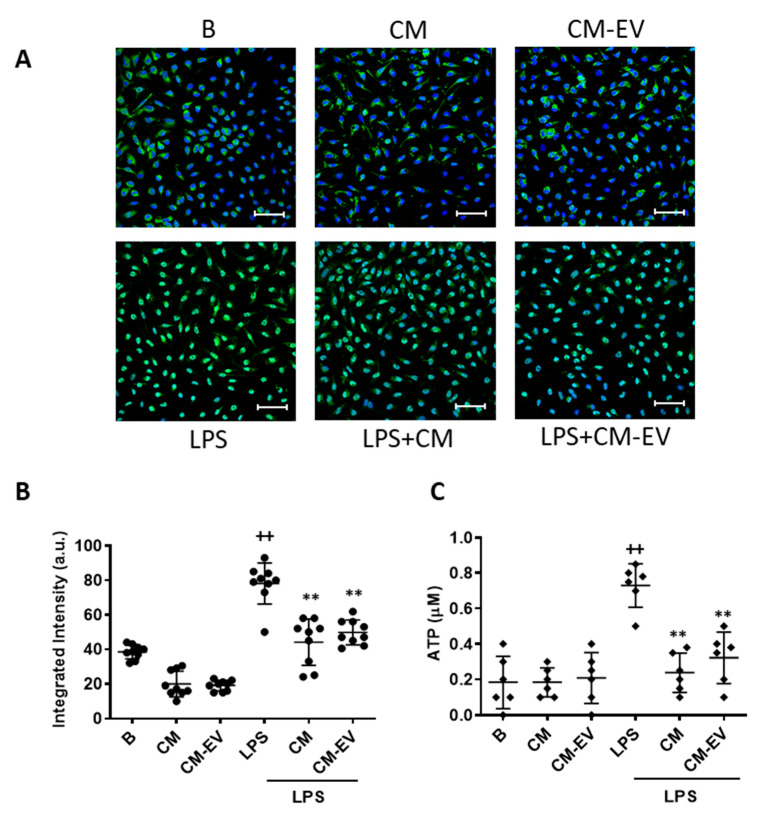
NF-κB nuclear translocation and ATP release. For NF-κB translocation, macrophages were incubated with CM or CM-EV for 30 min and immunofluorescence was determined by confocal microscopy using an NF-κB p65 XP^®^ Rabbit (Alexa Fluor^®^ 488 Conjugate). (**A**) Representative images. Microscopic magnification of the objective lens 40 x. Bar = 50 µm. (**B**) Quantification of nuclear translocation. For ATP release (**C**), macrophages were incubated with CM or CM-EV in the presence or absence of LPS for 20 h. ATP levels were determined by luminescence in the medium. B: cells incubated with control medium and not stimulated with LPS. Data are presented as mean ± SD (*n* = 6–9) from 3 independent experiments. One-way analysis of variance followed by Tukey’s post hoc test; ++ *p* < 0.001 versus B; ** *p* < 0.01 versus LPS control.

TLRs activate specific signaling cascades, such as the NF-κB pathway, to induce the transcription of pro-inflammatory genes [27]. The results of the previous experiments indicate that the anti-inflammatory properties of ASC CM are not due to the presence of EV. Therefore, we focused on CM and its active fraction CM-EV. As NF-κB is the main transcription factor regulating the expression of pro-inflammatory mediators in macrophages, we assessed its nuclear translocation by immunofluorescence to determine the effects of CM and CM-EV. LPS strongly induced the translocation of p65 into the nucleus (Figure 5A,B) which was significantly decreased in macrophages treated with either CM or CM-EV.

### 2.7. Adenosine Triphosphate (ATP) Release

ATP release plays a relevant role in the LPS-induced innate immune response and may contribute to the enhanced production of some pro-inflammatory mediators by activated macrophages [28]. As shown in Figure 5C, exposure of macrophages to LPS resulted in a significant ATP release compared to unstimulated control cells and this effect was reduced by the treatment with either CM or CM-EV.

## 3. Discussion

In this study, we have examined the immunomodulatory effects of ASC secretome on the innate immune response to determine the contribution of EV (EX and MV) and the soluble fraction (CM-EV). The regulation of macrophage activation by LPS is critical for controlling the initial phases of the immune response. Activation of TLR4 by LPS induces the expression of pro-inflammatory mediators which is downregulated by the secretome of mouse-derived ASC. We have shown that IL-1β, TNFα, and nitric oxide (NO) release induced by LPS stimulation of macrophages is strongly decreased by CM and CM-EV, whereas EX and MV only exerted a weak effect on TNFα. The IL-1 family of cytokines plays an important role in the innate response to infection but also induces the production of chemokines, cytokines, NO, degradative enzymes, etc., contributing to inflammation and tissue destruction [29,30]. A broad range of immune and inflammatory functions have also been ascribed to TNFα [31] and NO which have been implicated in sepsis, autoimmune conditions, etc., [32]. Therefore, the downregulation of these mediators could play an important role in the anti-inflammatory effects of CM which would be mainly mediated by its soluble fraction. It is known that LPS induces ATP release which stimulates NO and IL-1β production in mouse-derived macrophages [33,34]. Interestingly, we have shown that CM reduces ATP release induced by LPS which may contribute to the anti-inflammatory effects of ASC secretome.

Other macrophage responses were also differently affected by CM fractions. Thus, we have shown that the ASC secretome elicits pro-migration effects of neutrophils and monocytes which are not dependent on the EV fraction. The pro-inflammatory mediators released by macrophages during the early stage of inflammation induce an increase in the migration of immune cells. Neutrophils are the first cells to migrate within minutes to the focus of inflammation to phagocytose particles, release bactericidal molecules, cytokines, chemokines, and reactive oxygen species, and produce neutrophil extracellular traps. They also interact with other cells such as macrophages, dendritic cells, natural killer cells, T cells, and B cells, potentiating or downmodulating the innate and adaptive immunity [35]. Neutrophil migration seems to be necessary for normal healing of tissues although excessive tissue destruction leads to persistent inflammation preventing the progression to healing stages [36]. Some of the bioactive molecules secreted by MSC are chemokines, which are involved in the recruitment of different cells from the immune system. Our data indicate that CM from ASC enhances the migration of neutrophils in the presence of unstimulated macrophages which is likely due to the presence of neutrophil chemokines such as CXCL-1. Nevertheless, our results indicate that CM does not modulate the migratory capacity of neutrophils in the presence of LPS-stimulated macrophages suggesting the contribution to this response of other factors not regulated by CM.

Monocytes migrate from the bone marrow to the focus of inflammation to play a key role in tissue homeostasis and immunity. We have shown that CM and EV-depleted CM increase monocyte migration in the presence of LPS stimulation. This aligns with published results on the pro-migration effects on human monocytes of human ASC treated with TNF-α [37] suggesting that CM may augment the repair response [23,38]. Migration enhancement by CM or CM-EV treatment of macrophages in the presence of LPS may be related at least in part to the content of CCL-2 since this chemokine plays an important role in monocyte migration and its effects are potentiated by LPS [39].

Macrophages are activated during inflammation, increasing their phagocytic activity to remove the strange particles or bacteria that can damage our organism. Additionally, phagocytosis of apoptotic bodies and debris is necessary for the resolution of inflammation and immune regulation [40]. Many studies have demonstrated that MSC can regulate the activation and function of macrophages through the secretion of soluble factors or by cell-to-cell contact [41,42]. However, little is known about the influence of EV from MSC on the phagocytic activity of macrophages. In contrast to the inhibitory effect of CM, our results have shown that EV increase the phagocytic activity of inert particles by LPS-stimulated macrophages. These data are in line with the work of Monsel et al. [43] which reported increased monocyte phagocytosis by EV from MSC in a severe pneumonia mouse model.

TLR activation triggers signaling pathways inducing the expression of immune and pro-inflammatory genes. In many inflammatory conditions, there is an increased TLR expression leading to aberrant TLR activation [44]. On the contrary, TLR4 deficiency prevents the production of pro-inflammatory cytokines and the development of arthritis in experimental models [45]. To explore the mechanisms responsible for the anti-inflammatory effects of CM, TLR4, and CD14 protein expression was analyzed by flow cytometry. We have shown that CM and CM-EV, but not EV, downregulate TLR4 expression stimulated by LPS raising the possibility that the reduction in the TLR4 signaling pathway has a role in the anti-inflammatory activity of ASC secretome. LPS stimulation of macrophages through TLR4 activates NF-κB leading to the synthesis of pro-inflammatory mediators such as cytokines, chemokines, NO, etc., [46]. Our data indicate that the inhibition of NF-κB nuclear translocation by CM could mediate its anti-inflammatory effects in mouse-derived macrophages stimulated with LPS.

In conclusion, we have shown that the ASC secretome controls the early inflammatory response induced by LPS in murine macrophages and these effects are mediated by the soluble fraction. The data presented here gives insight into a regulatory pathway involving downregulation of TLR4 expression and NF-κB activation with relevance to the anti-inflammatory effects of murine ASC secretome. Although more studies are necessary for a complete characterization of the secretory profile of mouse-derived perigonadal ASC, these findings improve our understanding of ASC regulatory properties on the innate response.

## 4. Materials and Methods

### 4.1. Animals

Male CD1 mice (Janvier, Le Genest-Saint-Isle, France) between 8 and 10 weeks of age were used for all experiments. Mice were housed in plastic cages (four per cage) with wood chips and maintained in a quiet room at 21 ± 2 °C on a 12 h light–dark cycle. Standard diet and water were provided ad libitum. All experiments were performed following European regulations for the handling and use of laboratory animals (Directive 2010/63/EU and Spanish R. D. 53/2013). The protocols were approved by the Institutional Animal Care and Use Committee (Comité de Etica de Experimentación Animal de la Universidad de Valencia, Spain) and Generalitat Valenciana (number 2015VSC/PEA/00254, 12 January 2016).

### 4.2. Isolation of ASC and Preparation of CM

ASC were obtained from perigonadal adipose tissue of CD1 mice. Adipose tissue was minced and digested with 1 mg/mL of type IA collagenase (Sigma–Aldrich^®^, St. Louis, MO, USA) in Roswell Park Memorial Institute (RPMI) medium (Gibco^®^, Life Technologies, Madrid, Spain) supplemented with penicillin (100 U/mL) and streptomycin (100 μg/mL) (1% penicillin/streptomycin) (Sigma–Aldrich^®^, St. Louis, MO, USA) at 37 °C for 1 h and then centrifuged at 500× *g* for 10 min. After removal of the supernatant, the pellet (stromal vascular fraction) was resuspended in RPMI medium supplemented with 10% EV-free fetal bovine serum (FBS) (Biowest SAS, Nuaille, France), 1% penicillin/streptomycin, and filtered through a 70 μm membrane. Cells were seeded onto cell culture flasks and maintained at standard culture conditions (5% CO_2_ enriched atmosphere at 37 °C). 24 h later non-adherent cells were removed and the RPMI medium was changed by Dulbecco’s modified Eagle’s medium (DMEM) HAM’s F-12 medium (Sigma–Aldrich^®^, St. Louis, MO, USA) containing 15% EV-free FBS, 1% penicillin/streptomycin. To remove EV from FBS, serum was centrifuged for 18 h at 120,000× *g* and 4 °C using an SW-28 swinging-bucket rotor (Beckman Coulter, Brea, CA, USA). ASC were characterized phenotypically by flow cytometry (Becton Dickinson LSRFortessaTM, BD Biosciences, Franklin Lakes, NJ, USA), using positive markers anti-CD105-Phycoerythrin (PE) and anti-CD29-Peridinin-Chlorophyll protein (PerCP)-eFluorTM 710 and negative markers anti-CD11b-Allophycocyanin (APC) and anti-CD45-Fluorescein isothiocyanate (FITC) (eBioscienceTM, San Diego, CA, USA). CM was obtained by centrifugation of the supernatant of cultured ASC at passage 1 every 48–72 h and then was stored at −80 °C in sterile conditions.

### 4.3. Isolation and Culture of Peritoneal Macrophages

Resident macrophages were isolated from the peritoneal cavity of CD1 mice. A volume of 10 mL of PBS was injected into the peritoneal cavity. Peritoneal exudate cells were collected in a 50 mL conical tube and centrifuged at 500× *g*, for 6 min. Then, the pellet was resuspended in lysis buffer (Sigma–Aldrich^®^, St. Louis, MO, USA) for 30 s to eliminate erythrocytes. The cell pellet was rinsed with RPMI medium and cells were seeded at 3 × 10^5^ cells/well in 500 μL of RPMI supplemented with 10% FBS and 1% penicillin/streptomycin on a 24-well plate. Cells were maintained at standard culture conditions (5% CO_2_ enriched atmosphere at 37 °C). After 24 h the medium was changed to DMEM HAM’s F-12 medium with 10% FBS and 1% penicillin/streptomycin. Before beginning each experiment, the medium from macrophages was replaced with DMEM HAM’S F-12 medium supplemented with 10% EV-depleted FBS and 1% penicillin/streptomycin. Macrophages were characterized phenotypically by flow cytometry using positive markers anti-CD11b-APC and anti-F4/80-PerCP-Cy(Cyanine)5.5. To perform the experiments, macrophages were stimulated with LPS from Escherichia coli 0111:B4 (Sigma–Aldrich^®^, St. Louis, MO, USA, 1 µg/mL) at different times. To measure the expression of TLR4 and CD14 on the surface of macrophages, anti-TLR4-PE (BioLegend^®^, San Diego, CA, USA) and anti-CD14-PE (eBioscience^TM^, San Diego, CA, USA) antibodies were used besides an isotype control.

### 4.4. Isolation and Culture of Neutrophils and Monocytes

Neutrophils were isolated from CD1 mice femur and tibiae bone marrow by flushing PBS through a 10 mL syringe and a 25 G needle. The bone marrow suspension was filtered through a 70 μm filter. Then, the cell suspension was centrifuged at 500× *g* for 5 min at 23 °C. Neutrophils were isolated by immunomagnetic cell sorting (positive selection) using the antibody anti-Ly-6G-Gr1-FITC (eBioscience^TM^, San Diego, CA, USA) and anti-FITC conjugated with microbeads according to the manufacturer’s instructions (Miltenyi Biotec, Madrid, Spain). For neutrophil characterization by flow cytometry (Becton Dickinson LSRFortessa^TM^), cells were stained with positive markers anti-CD11b-APC and anti-Ly6G-FITC.

To isolate monocytes, bone marrow from femurs and tibiae of mice were harvested in a conical tube by flushing PBS through a 10 mL syringe and a 25 G needle. After removing erythrocytes with lysis buffer (Sigma–Aldrich^®^, St. Louis, MO, USA), cells were plated on a 100-mm diameter tissue culture dish and incubated at 37 °C and 5% CO_2_ for 3 h. Then, cells were harvested with trypsin 0.25% followed by several washes with DMEM HAM’s F-12 medium and used to perform the migration assay. Monocytes were characterized by flow cytometry (Becton Dickinson LSRFortessa^TM^) using positive markers anti-CD11b-APC and anti-Ly6C, and negative marker anti-F4/80-PerCP-Cy5.5.

### 4.5. Isolation of EV from ASC CM

The method used to isolate EV from CM of ASC was differential centrifugation combined with size filtration. CM was centrifuged at 300× *g* for 10 min to remove cellular debris and then filtered through a 0.8 μm filter (Merck, Darmstadt, Germany) by hydrostatic pressure and centrifuged at 12,600× *g* for 30 min at 4 °C to pellet the MV. Next, to obtain the EX, the supernatant was filtered through a 0.22 μm filter (Merck, Darmstadt, Germany) and then ultracentrifuged at 100,000× *g* for 70 min at 4 °C (Beckman Coulter, Brea, CA, USA). The pellets containing MV or EX were washed several times with PBS and finally resuspended in 15 μL of PBS and stored at −80 °C until use. The supernatant obtained after the ultracentrifugation (EV-free CM) was stored at −80 °C for further experiments.

TRPS and TEM. The size distribution and concentration of samples of MV or EX were determined by TRPS (qNano, Izon Science Ltd., Oxford, UK) using NP300 and NP150 nanopore membranes. The single calibration sample was performed with carboxylated polystyrene particles of known concentration and particle size, CPC400 and CPC200 (Izon Science Ltd., Oxford, UK) according to the manufacturer. At least 500 events/sample were counted. The morphology characterization was performed using TEM by the Microscopy Service (SCSIE, University of Valencia, Valencia, Spain). The pellets of MV or EX were fixed in Karnovsky’s solution. After rinsing the samples with PBS and post-fixed in osmium tetroxide, they were dehydrated in graded ethanol and embedded in LR-White resin. Following overnight polymerization of samples at 60 °C, resin blocks were cut with the ultramicrotome Ultracut UC6 (Leica, Wetzlar, Germany). The ultrathin sections (60 nm) obtained were contrasted with uranyl acetate 2% for 25 min and lead citrate 3% for another 12 min and observed using Jeol JEM-1010 transmission electron microscope (JEOL Ltd., Tokyo, Japan) at 80 kV. Images were acquired with a digital camera MegaView III with Olympus Image Analysis Software (Olympus, Tokyo, Japan).

### 4.6. MTT Assay

The mitochondrial reduction in MTT to formazan as an indicator of cell viability was assayed in macrophages treated with MV, EX, CM or EV-free CM in the presence or absence of LPS (1 μg/mL) in 24-well plates for 20 h. Then, cells were incubated with MTT (200 μg/mL) for 90 min at 37 °C in the dark. After removing the medium, cells were solubilized in dimethyl sulfoxide (150 μL) to quantitate formazan at 490 nm in the spectrophotometer Victor3 microplate reader (PerkinElmer, Madrid, Spain).

### 4.7. Enzyme-Linked Immunosorbent Assay

Macrophages were incubated with MV (9 × 10^4^ particles/mL), EX (2 × 10^7^ particles/mL), CM (0.5 mL) or EV-free CM (0.5 mL) in the presence or absence of LPS (1 µg/mL) for 4 h or 20 h. TNFα, IL-1β, CXCL-1 and CCL-2 were measured in supernatants with ELISA kits with a sensitivity of 31.2 pg/mL for TNFα, 15.6 pg/mL for IL-1β (R&D Systems, Minneapolis, MN, USA), 8.0 pg/mL for CXCL-1 (PromoCell GmbH, Heidelberg, Germany) and 2.74 pg/mL for CCL-2 (RayBiotech, Norcross, GA, USA) according to the manufacturer’s instructions using a Victor3 microplate reader (PerkinElmer).

### 4.8. Determination of Nitric Oxide

Macrophages were incubated with MV (9 × 10^4^ particles/mL), EX (2 × 10^7^ particles/mL), CM (0.5 mL) or EV-free CM (0.5 mL) in the presence or absence of LPS (1 µg/mL) for 4 h and 20 h. Supernatants were used to measure NO production by fluorometric determination of nitrite levels [47] using a Victor3 microplate reader (PerkinElmer, Waltham, MA, USA).

### 4.9. Cell Migration

Cell migration was performed using a transwell system (6.5 mm-diameter polycarbonate membranes with 3 or 5 μm-diameter pores; Corning, Glendale, AZ, USA). Macrophages were seeded in the lower compartment whereas neutrophils or monocytes were added to the upper compartment. Resident peritoneal macrophages were seeded at 3 × 10^5^ cells/well in 500 μL of RPMI medium supplemented with 10% FBS and 1% penicillin/streptomycin in the lower compartment of the transwell in a 24-well plate. Cells were maintained at 37 °C and 5% CO_2_. Macrophages were incubated with CM, CM-EV, EX or MV in the presence or absence of LPS (1 μg/mL), as previously described. Then, 20 h later, 5 × 10^5^ neutrophils or monocytes were added to the upper compartment of the transwell (insert) and migration proceeded for 4 h. After this time, migrated cells and macrophages were harvested from the lower compartment, and the number of migrated cells was quantified using a flow cytometer (Becton Dickinson LSRFortessa^TM^). The supernatants were collected to determine chemokine levels by ELISA.

### 4.10. Phagocytosis

For flow cytometry, macrophages (3 × 10^5^ cells/well) were incubated with EX (2 × 10^7^ particles/mL), MV (9 × 10^4^ particles/mL), CM (0.5 mL), or EV-free CM (0.5 mL) in the presence or absence of LPS (1 μg/mL) for 20 h. Then, 10 fluorescent polystyrene beads/cell (FluoSpheres^®^, Molecular Probes Thermo Fisher Scientific, Rochester, NY, USA) were added, and the phagocytosis process proceeded for 4 h. Phagocytosis was stopped by adding cold PBS into the wells or putting the plate on ice for 30 s. Then, cells were washed with PBS, fixed with paraformaldehyde, and phagocytic cells were determined by flow cytometry (Becton Dickinson LSRFortessa^TM^).

### 4.11. Immunofluorescence

Resident peritoneal macrophages were plated on an eight-well Lab-Tek chamber slide (Thermo Fisher Scientific) in an incubator at standard conditions in RPMI medium supplemented with 10% FBS and 1% penicillin/streptomycin. After 24 h the medium was changed to DMEM HAM’s F-12 medium with 10% FBS and 1% penicillin/streptomycin. Before the experiment, the medium was replaced by DMEM HAM’S F-12 medium supplemented with 10% EV-depleted FBS and 1% penicillin/streptomycin. Macrophages were incubated with CM and EV-free CM in the presence or absence of LPS (1 μg/mL) for 30 min. Cells were washed with PBS and fixed with the use of 4% p-formaldehyde diluted in PBS for 15 min at room temperature. After washing with PBS, Blocking Buffer was added for 1 h at room temperature and then cells were incubated with the monoclonal antibody against NF-κB p65 XP^®^ Rabbit (Alexa Fluor^®^ 488 Conjugate) (Cell Signaling Technology, Danvers, MA, USA) overnight at 4 °C. Slides were mounted in ProLong Gold antifade reagent with 4′,6-diamidino-2-phenylindole (DAPI) (Molecular Probes Thermo Fisher Scientific) and observed in a confocal microscope (Olympus FV1000, Tokyo, Japan). Quantification of the immunofluorescence image was performed with CellProfiler software (version 4.0.6, Broad Institute, Cambridge, MA, USA) on 40× images.

### 4.12. Determination of ATP Concentration

Mouse-derived macrophages were seeded onto 96-well plates at a concentration of 450,000 cells/mL. After removing the nonadherent cells, 100 μL of CM or EV-free CM in DMEM HAM’S F-12 supplemented with 10% EV-depleted FBS medium and 1% penicillin/streptomycin were added into the wells for 20 h in the presence or absence of 1 μg/mL LPS. Then, ATP concentration was measured in the supernatant of macrophages, using the ATPlite Luminescence Assay System according to the manufacturer’s instructions (PerkinElmer).

### 4.13. Statistical Analysis

Experimental data were analyzed using the GraphPad Prism 7.03 software (GraphPad Software, La Jolla, CA, USA). The level of statistical significance was determined by using the one-way analysis of variance followed by Tukey’s post hoc test. A *p*-value of less than 0.05 was considered to indicate statistical significance.

## Data Availability

The data presented in this study are available on request from the corresponding author.
